# Upregulation of tumor necrosis factor receptor-associated factor 6 correlated with synovitis severity in rheumatoid arthritis

**DOI:** 10.1186/ar3866

**Published:** 2012-06-04

**Authors:** Lang-Jing Zhu, Lie Dai, Dong-Hui Zheng, Ying-Qian Mo, Xia Ou-Yang, Xiu-Ning Wei, Jun Shen, Bai-Yu Zhang

**Affiliations:** 1 Department of Rheumatology, Sun Yat-Sen Memorial Hospital, Sun Yat-Sen University, Guangzhou, 510120, P.R China; 2 Department of Radiology, Sun Yat-Sen Memorial Hospital, Sun Yat-Sen University, Guangzhou, 510120, PR. China

## Abstract

**Introduction:**

Rheumatoid arthritis (RA) is a chronic inflammatory disease leading to joint destruction and disability. Focal bone erosion is due to excess bone resorption of osteoclasts. Tumor necrosis factor receptor-associated factor 6 (TRAF6) is one of the critical mediators both in inflammatory signal pathway and differentiation and resorption activity of osteoclasts. Here we aimed to investigate TRAF6 expression in RA synovium and its correlation with histological synovitis severity and radiological joint destruction in RA.

**Methods:**

Synovitis score was determined in needle biopsied synovium from 44 patients with active RA. Synovium from nine patients with osteoarthritis (OA) and seven with orthopedic arthropathies (Orth.A) were enrolled as "less inflamed" disease controls. Serial sections were stained immunohistochemically for TRAF6 as well as CD68 (macrophage), CD3 (T cell), CD20 (B cell), CD38 (plasmocyte), CD79a (B lineage cells from pre-B cell to plasmocyte stage), and CD34 (endothelial cell). Double immunofluorescence staining of TRAF6 and CD68 were tested. Densities of positive staining cells were determined and correlated with histological disease activity (synovitis score) and radiographic joint destruction (Sharp score).

**Results:**

TRAF6 expression was found in the intimal and subintimal area of RA synovium, with intense staining found in the endochylema and nucleus of intimal synoviocytes and subintimal inflammatory cells. Double immunofluorescence staining showed TRAF6 was expressed in most of the intimal cells and obviously expressed in CD68+ cells and some other CD68- cells in subintimal area. Synovial TRAF6 was significantly over-expressed in the RA group compared with the OA and Orth.A group (2.53 ± 0.94 vs. 0.72 ± 0.44 and 0.71 ± 0.49, *P *< 0.0001). Synovial TRAF6 expression in RA correlated significantly with synovitis score (r = 0.412, *P *= 0.006), as well as the inflammatory cell infiltration (r = 0.367, *P *= 0.014). Significant correlation was detected between synovial TRAF6 expression and intimal CD68+ cells, as well as the cell density of subintimal CD68+ cells, CD3+ cells, CD20+ cells, CD38+ cells, and CD79a+ cells (all *P *< 0.05).

**Conclusions:**

Elevated synovial TRAF6 expression correlated with synovitis severity and CD68+ cell density in RA. It is, therefore, hypothesized that synovial TRAF6 is involved in the pathogenesis of synovial inflammation and osteoclast differentiation in RA.

## Introduction

Rheumatoid arthritis (RA) is a chronic autoimmune disease characterized by chronic inﬂammatory synovitis, leading to invasion of synovial tissue into the adjacent cartilage matrix with degradation of articular cartilage and bone, which constitutes a major cause of progressive disability and crippling of RA patients [1]. Persistent synovial inflammation is one of the most characteristic features of RA, which leads to cartilage and bone destruction, and subsequent disability in RA [2-4]. The pathogenesis of RA is complex and encompasses many cell types, including T cells, B cells, monocytes/macrophages and fibroblast-like synoviocytes (FLSs); each has been proved to play distinct, complex and interrelated roles in the pathogenesis and progression of RA [5,6]. Our previous study found synovial infiltration with CD79a+ B cells, but not other B cell lineage, correlated with synovitis score and joint destruction in RA, which indicated synovial CD79a+ B cells may be a helpful biomarker for histologic disease activity and involved in the pathogenesis of joint destruction in RA [7]. However, the related mechanism was not clear. Recent study showed that increased osteoclast formation and activity contributes to local and systemic abnormalities of bone remodeling, including bone erosion as well as focal and systemic osteoporosis [8]. Several studies have shown that osteoclast precursors and mature osteoclasts were abundant at sites of arthritic bone erosion [9,10].

A tumor necrosis factor (TNF)-family molecule receptor activator of nuclear factor κB (RANK) ligand (RANKL), and its receptor RANK, are known as the major regulators for the differentiation and activation of osteoclasts [11,12]. It has been shown that both RANKL and RANK were expressed on cells of RA synovium [13]. Signaling occurs following the association of RANK and RANKL primarily through tumor necrosis factor receptor-associated factor 6 (TRAF6), which influences the differentiation of osteoclasts through several pathways, including nuclear factor κB (NF-κB) and mitogen-activated protein (MAP) kinases [14]. TRAF6 seems to be critical for RANK signaling in osteoclasts since genetical deficiency of TRAF6 resulted in compromised diﬀerentiation and defective activation of osteoclasts [15]. Furthermore, results from reconstitution experiments where *rank*^-/- ^cells were manipulated to express RANK mutants lacking the TRAF6-binding region provided evidence for the involvement of TRAF6 in RANK-directed cytoskeletal organization and resorption function of osteoclasts [16].

TRAF6 is a member of the group of seven closely related TRAF proteins, which serve as an adapter coupling the TNF receptor (TNFR) superfamily to intracellular signaling events. Moreover, TRAF6 is unique for signaling downstream of another receptor family, the interleukin-1 (IL-1) receptor/Toll-like receptor (IL-1R/TLR) superfamily, which plays critical roles in inflammation, innate and adaptive immune responses [16]. TRAF6 has been proved as a key adapter in regulating various signaling pathways of inflammatory response and a diverse array of physiological processes, including adaptive immunity, innate immunity and bone metabolism, as well as development of several tissues including lymph nodes, mammary glands, skin and central nervous system [17,18].

RA is one of the most severe chronic joint diseases by virtue of persistent synovial inflammation and destruction of cartilage and bone. TRAF6 is critical for inflammatory signaling pathways, as well as RANK signaling in diﬀerentiation and activation of osteoclasts. It has been reported that TRAF6 was up-regulated in basal RA-FLS compared to osteoarthritis (OA)-FLS, and lipopolysaccharide (LPS) stimulation could induce the up-regulation of TRAF6 in RA-FLS [19]. To date, TRAF6 expression in RA synovium has not been examined. The pathophysiological role of TRAF6 in RA has been poorly understood and whether TRAF6 is the linkage of CD79a+ B cells and inflammation/joint destruction in RA has not been clarified. Therefore, we examined TRAF6 expression in RA synovium and analyzed its correlation with clinical disease activity, histological synovitis severity and radiological joint destruction.

## Materials and methods

### Patients

Forty-four Chinese RA patients, who fulfilled the American College of Rheumatology (ACR) 1987 criteria for RA [20], or the 2010 RA classification criteria [21], were recruited from the Department of Rheumatology of Sun Yat-Sen Memorial Hospital, Sun Yat-Sen University in Guangzhou, P.R China. All patients had active disease, defined as a Disease Activity Score 28-joint assessment (DAS28) > 3.2. Eleven of them were newly diagnosed and were naïve to any treatment with prednisone or disease modifying antirheumatic drugs (DMARDs). Synovium from nine patients with OA as defined according to established clinical criteria, and seven with orthopedic arthropathies (Orth.A, consisting of femur fracture (*n *= 1), meniscus injury (*n *= 3), meniscus cyst (*n *= 1), obsolete knee joint injury (*n *= 1), and plica syndrome of the knee joint (*n *= 1)) were recruited from the Department of Orthopedics as "less inflamed" disease control [22]. The demographic and drug treatment data of the RA patients are shown in Table 1. All participants provided informed consent. The study was approved by the ethic committee of Sun Yat-Sen Memorial Hospital, Sun Yat-Sen University, and was performed in accordance with the Helsinki Declaration.

**Table 1:** Baseline characteristics of the 44 RA patients^▲^

	RA (*n *= 44)
***Demographic characteristics***	
Age, years, median (range)	51 (23 to 75)
Female, n (%)	34 (81)
***Disease status***	
Disease duration, months, median (range)	36 (1 to 480)
RF positive, n (%)	35 (80)
ACPA positive, n (%)	37 (84)
DAS28, median (range)	5.84 (3.22 to 7.92)
Synovitis score^△^, median (range)	4 (1 to 6.5)
***Medications***	
Corticosteroids, n. (%)	27 (61)
Methotrexate, n (%)	16 (36)
Leflunomide, n (%)	10 (23)
Sulfasalazine, n (%)	2 (5)
Hydroxychloroquine, n (%)	1 (2)
Etanercept, n (%)	3 (7)

▲ ACPA, anti-cyclic citrullinated peptide antibody; DAS28, Disease Activity Score 28-joint assessment; n, number of patients; RA, rheumatoid arthritis; RF, rheumatoid factor; SD, standard deviation.

△ Synovitis score according to Krenn *et al. *[19,22,23], based on the following basic morphological parameters of synovitis: (a) hyperplasia of intima; (b) cellular density of synovial stroma and (c) inflammatory cell infiltration, including fibroblasts, endothelial cells, histiocytes, macrophages, and multinucleated giant cells. All parameters are graded from 0 (absent), 1 (slight), 2 (moderate) to 3 (strongly positive). The values of all parameters are summarized, resulting in a final score between 0 and 9 [22].

### Synovitis assessment

Synovium from inflamed knees of the recruited RA patients was collected by closed Parker-Pearson needle biopsy [23]. At least six pieces of synovial tissues were obtained per patient to minimize sampling error [24]. The OA and Orth.A specimens were obtained by knee arthroplasty or arthroscopy. Samples were fixed in 10% neutral formalin, embedded in paraffin, cut in microsections of 5 μm and stained with hematoxylin and eosin (H&E) according to routine procedures.

Only tissue pieces containing synovial intima and vascularized subintima were included in the analyses. At least three such pieces were evaluated for each specimen. Histologic changes in H&E-stained sections were graded by two observers (L-J Zhu and Y-Q Mo) according to a previously validated synovitis score [22,25,26], with the modification that the average of all fields containing synovial intima was recorded per specimen.

Three features of chronic synovitis (hyperplasia of intima, cellular density of synovial stroma, inflammatory cell infiltration) were scored from 0 to 3, with the sum providing the synovitis score, which was interpreted as follows: 0 to 1, no synovitis; 2 to 4, low-grade synovitis; 5 to 9, high-grade synovitis. This score correlates positively with synovial proliferation and expression of the CD68 antigen, a well established synovial tissue biomarker for RA [22].

### Immunohistochemistry

Serial sections (5 μm thickness) from paraffin blocks were stained by commercial antibody preparations according to standard staining protocols in a three-step immunoperoxidase method. Rabbit anti-human TRAF6 monoclonal antibody (mAb) (EP591Y, Abcam plc. Cambridge Science Park, Cambridge, UK) (concentration of the stock solution is not stated), mouse monoclonal antibodies (Invitrogen Corporation, San Diego, CA, USA) of CD68 (macrophages; clone KP-1, concentration of the stock solution is 0.04 mg/ml), CD3 (T cells; clone PS1, concentration of the stock solution is 0.2 mg/ml), CD20 (B cells; clone L26, concentration of the stock solution is 0.1 mg/ml), CD38 (plasma cells; clone SPC32, concentration of the stock solution is not stated), and CD34 (vascular endothelial cells; clone QBEnd/10, concentration of the stock solution is not stated), as well as rabbit monoclonal antibody of CD79a (B lineage cells from pre-B cell to plasmocyte stage; clone SP18, concentration of the stock solution is not stated) were used. Sections were deparaffinized with xylene, ethanol and demineralized water. Antigens were then retrieved by boiling in 1 mM EDTA (pH 8.0) for 15 to approximately 20 minutes. After the sections had been washed in demineralized water and phosphate buffered saline (PBS), the primary antibody diluted properly (TRAF6 at a 1/50 dilution, CD68 at a 1/200 dilution, CD3 at a 1/50 dilution, CD20 at a 1/40 dilution, CD38 at a 1/50 dilution, CD34 at a 1/100 dilution, and CD79a at a 1/100 dilution) was added and incubated overnight at 4°C. After washing with PBS, the sections were incubated with EnVision Mouse or Rabbit conjugate (Dako Corporation, Carpinteria, California, USA) for 15 minutes at 37°C. The color reaction was completed with the DAB+ substrate. Sections were counterstained with hematoxylin. Nonspecific isotype IgG was used as a negative control. Absence of staining due to technical failure was excluded by including appropriate positive control tissues in each staining run.

The densities of CD79a, CD20, CD38, CD3 and CD68 positive staining cells as well as microvascular count (MCV, confirmed by CD34+ endothelial cell) were determined by manual counting. A selection of 17 high-power field (hpf) (400×) in the superficial subintima were examined for each specimen [27]. A 1-mm graticule was used in each hpf with its edge placed just below the intima. The measured value per hpf was converted to the value per square millimeter (mm^2^) by using the conversion factor × 0.0625^-1 ^[28]. Semiquantitative analysis was performed to evaluate the intensity of intimal CD68+ macrophage-like synoviocytes of RA synovium as previously described [29]. Intimal CD68+ cells were scored on a scale of 0 to 4. A score of 0 represented no or minimal infiltration, while a score of 4 represented intense infiltration. Synovial expression of TRAF6 was scored semi-quantitatively on a five-point scale (0: absent, 1: 1% to 25%, 2: 26% to 50%, 3: 51% to 75%, 4: 76% to 100%) as described previously [30]. A synovial TRAF6 score ≤ 2 is considered as low expression, and > 2 as high expression. Each specimen was scored by two independent observers (L-J Zhu and Y-Q Mo) in a random order; differences between the observers were resolved by mutual agreement, the kappa statistic quantifying the agreement between them was 0.920 to 0.946.

### Immunofluorescence staining

For double immunofluorescence staining of CD68 and TRAF6, paraffin-embedded sections were deparaffinized with xylene, ethanol and demineralized water. Nonspecific binding was blocked with 5% bovine serum albumin in PBS, and then the sections were incubated overnight at 4°C with rabbit anti-TRAF6 mAb (at a 1/30 dilution) or normal rabbit IgG (control), and mouse anti-CD68 mAb (at a 1/100 dilution). The samples were then incubated with Alexa Flour 633-conjugated goat anti-rabbit IgG and Alexa Fluor 488-conjugated goat anti-mouse IgG at a 1/1,000 dilution (Invitrogen Corporation, both of the concentrations of the stock solutions are 2 mg/ml) for one hour at room temperature. DAPI (Sigma-Aldrich, St. Louis, MO, USA) was subsequently used for nuclei staining for five minutes and cover slips were mounted using ProLong^® ^Gold Antifade Reagent (P36934, Invitrogen Corporation). Images were analyzed and collected with 160 Zeiss LSM 510 Confocal Imaging System (Zeiss, Jena, Germany).

### Disease assessments

The disease activity of all RA patients was assessed at the time of recruitment by DAS28-C-reactive protein (CRP), which was calculated as the following formulas [31]: DAS28-CRP = (0.56 × sqrt(28TJC) + 0.28 × sqrt(28 swollen joint count (SJC)) + 0.36 × ln(CRP + 1)) × 1.1 + 1.15, where 28TJC represents the tender joint count of 28 joints, 28SJC represents the swollen joint count of 28 joints and CRP represents C-reactive protein. The value of DAS28-CRP between 2.6 and 3.2 is considered as low disease activity, a value between 3.2 and 5.1 as moderate activity, a value > 5.1 as high activity, and a value < 2.6 as remission. Disability status was reported by the patients using the Swedish version of the Stanford Health Assessment Questionnaire (HAQ) [32]. Other clinical and biological parameters were also collected: morning stiffness, gripping power, erythrocyte sedimentation rate (ESR), rheumatoid factor (RF) and anti-cyclic citrullinated peptide antibody (ACPA).

Radiographs of both hands/wrists (anteroposterior view) of each patient were obtained at the recruitment visit and the joint damage was assessed as a Sharp score [33,34]. Seventeen areas for erosion and 18 for joint space narrowing were assessed in each hand/wrist. The maximum erosion subscore per single joint is 5, and the maximum joint space narrowing subscore per single joint is 4, with the sum of both subscores constituting the total Sharp score. Radiographs were scored by one experienced radiologist (J Shen), who was not aware of any clinical or histological information. In a subgroup of patients, radiographs were reassessed by the same observer within two to four weeks. The correlation between these measurements was high (intraclass correlation coefficient 0.953 to 0.977).

### Statistical analysis

The statistical analysis was performed using SPSS for Windows 13.0 statistical software (SPSS Inc., Chicago, IL, USA). Data are presented as frequencies and percentages for categorical variables and mean ± SD for continuous variables, unless otherwise indicated. Because the expression of TRAF6 in synovium was not distributed normally, non-parametric testing using the Mann-Whitney rank sum test between two groups, or Kruskal-Wallis one way analysis of variance on ranks among three or more groups for continuous variables were used. Wilcoxon Signed Ranks Test was used for assessing the difference between baseline and 12 months after treatments. For assessing the correlation between synovial expression of TRAF6 and the histological or clinical parameters, Spearman's rank order correlation test was used. All significance tests were two-tailed and were conducted at the 5% significance level.

## Results

### Characteristics of the study patients

Demographic and clinical characteristics of all RA patients are shown in Table 1. Age, gender or disease duration did not differ among the patients with RA, OA and Orth.A. Ten of the RA patients were male and 34 were female. The mean age was 51.5 (range 23 to 75) years. Mean disease duration was 80.0 (range, 1 to 480) months. 80% (35/44) of the RA patients had positive RF, and positive ACPA was detected in 84% (37/44) of the RA patients. All RA patients had DAS28-CRP values > 3.2, suggesting active disease. Mean DAS28-CRP score was 5.85 (range 3.22 to 7.92). 75% (33/44) of the patients were in the high activity group, 25% (11/44) were in the moderate. A total of 25% (11/44) of the RA patients had never been treated with corticosteroids or DMARDs, except for Chinese herbs and/or pain-killers to relief arthralgia. A total of 27% (12/44) had taken corticosteroids alone before being referred to our hospital. A total of 41% (18/44) had taken one or more DMARDs, including methotrexate, leflunomide, sulfasalazine or hydroxychloroquine, while 32% (14/44) patients were actively on DMARDs and corticosteroids. Only 7% (3/44) had been treated with TNF-α blocker (etanercept). There was no washout period before synovial biopsy.

### Synovial expression of TRAF6 in RA, OA and Orth.A

**Figure 1:**
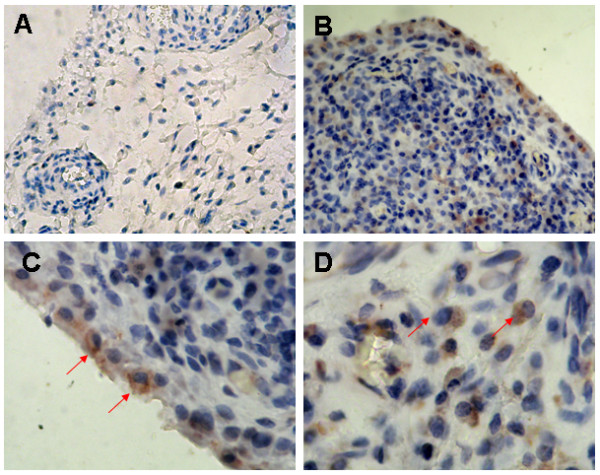
**Representative immunohistochemical findings of synovial TRAF6 expression**. **A**, Mild synovial TRAF6 expression in an OA patient; **B**, Intensive synovial TRAF6 expression in a RA patient; **C**, Expression of TRAF6 in intimal layer of the synovium in a RA patient. The arrows point to TRAF6+ synoviocytes located at intimal layer of the synovium; **D**, Expression of TRAF6 in subintimal layer of the synovium in a RA patient. The arrows point to TRAF6+ plasmocytes located at the subintimal layer of the synovium. (Anti-TRAF6 immunostaining, original magnification: A and B ×100, C and D ×400. OA, osteoarthritis; RA, rheumatoid arthritis; TRAF6, Tumor necrosis factor receptor-associated factor 6).

**Figure 2:**
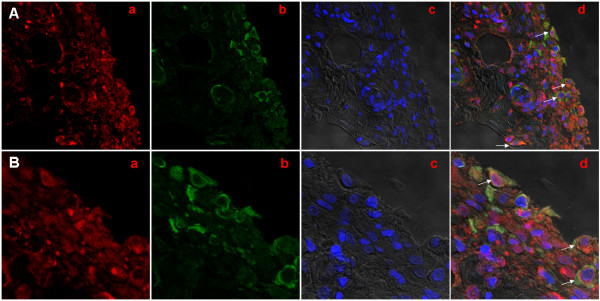
**Representative confocal microscopic images showed TRAF6 and CD68 by indirect double immunofluorescence staining in RA synovium**. **A**, original magnification, ×400; **B**, original magnification ×1,200. (a, TRAF6 (red); b, CD68 (green); c, DAPI (blue); d, merged a, b with c. The white arrows point to intimal and subintimal CD68/TRAF6 double+ cells. DAPI, diamidino-phenyl-indole; RA, rheumatoid arthritis; TRAF6, Tumor necrosis factor receptor-associated factor 6).

**Figure 3:**
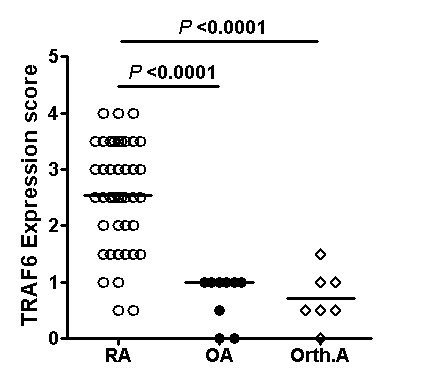
**Synovial expression of TRAF6 in RA, OA and Orth.A**. Scatterplots showing synovial TRAF6 expression scores in RA, OA and Orth.A patients as measured by immunohistochemistry. Synovial expression of TRAF6 was scored semiquantitatively on a five-point scale [29]. Each circle represents the determination for an individual with open circles indicating RA patients (*n *= 44), filled circles indicating OA patients (*n *= 9), and open diamonds indicating Orth.A patients (*n *= 7). Horizontal lines show the median for each population examined. The *P-*values were calculated using the Kruskal-Wallis one way analysis. (OA, osteoarthritis; Orth.A, orthopedic arthropathies; RA, rheumatoid arthritis; TRAF6, Tumor necrosis factor receptor-associated factor 6).

To explore whether synovial expression of TRAF6 was aberrant in RA patients, we determined synovial expression of TRAF6 in 44 RA patients with a blinded manner compared with 9 patients with OA and 7 with Orth.A as "less inflamed" disease control. TRAF6 expression was found in the intimal and subintimal layer of RA synovium, with intense staining found in the endochylema as well as nucleus of intimal synoviocytes and subintimal inflammatory cells (Figure 1). Double immunofluorescence staining showed TRAF6 was expressed in most of the intimal cells and obviously expressed in CD68+ cells and some other CD68- cells in the subintimal area (Figure 2). Significantly enhanced synovial expression of TRAF6 was detected from RA patients compared to those from OA or Orth.A patients (2.53 ± 0.94 vs 0.72 ± 0.44 and 0.71 ± 0.49, *P *< 0.0001) (Figure 3). There was no significant difference in synovial TRAF6 expression between OA and Orth.A patients.

In the RA group, 34.1% (15/44) patients showed low synovial TRAF6 expression, 65.9% (29/44) showed high synovial TRAF6 expression, and the mean (range) synovial TRAF6 expression score was 1.5 (0.0 to 2.0) and 3.1 (2.0 to 4.0), respectively.

### Correlation of synovial TRAF6 expression with the synovitis score

**Figure 4:**
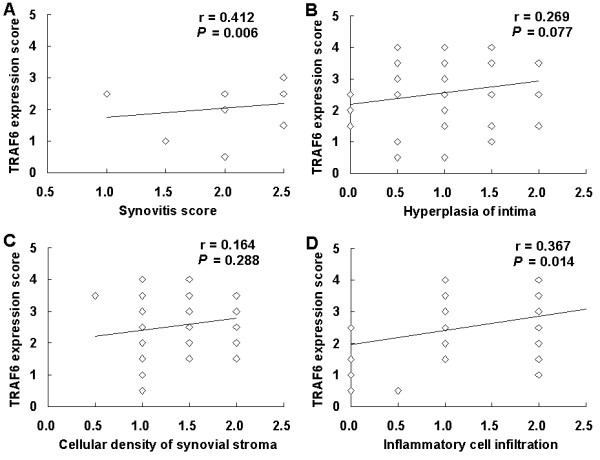
**Spearman's rank correlation analysis for synovial TRAF6 expression and synovitis score in RA**. **A**, Correlation between synovial TRAF6 expression and synovitis score; **B**, Correlation between synovial TRAF6 expression and hyperplasia of intima; **C**, Correlation between synovial TRAF6 expression and cellular density of synovial stroma; **D**, Correlation between synovial TRAF6 expression and inflammatory cell infiltration. (RA, rheumatoid arthritis; TRAF6, Tumor necrosis factor receptor-associated factor 6; *n *= 44).

Synovitis score was assessed in all 44 RA patients and the mean (range) score was 3.7 (1.0 to 6.5). 63.6% (28/44) patients had low-grade synovitis, 34.1% (15/44) had high-grade synovitis and 2.3% (1/44) patient had no synovitis. The mean synovial TRAF6 expression score in all RA patients was 2.5 (range 0.5 to 4.0). There was no significant difference in synovial TRAF6 expression between low-grade and high-grade synovitis group (2.4 ± 1.0 vs 2.7 ± 0.9, *P *= 0.320). Moreover, synovitis score was not significantly different between low and high synovial TRAF6 expression group (3.3 ± 1.4 vs 3.9 ± 1.2, *P *= 0.124). However, a significant correlation was found between synovial TRAF6 expression and synovitis scores from all 44 RA patients (r = 0.412, *P *= 0.006) (Table 2 and Figure 4A). When the three components of synovitis score (hyperplasia of intima, cellular density of synovial stroma and inflammatory cell infiltration) were subanalyzed, the inflammatory cell infiltration was found to correlate best with synovial TRAF6 expression (r = 0.367, *P *= 0.014) (Table 2 and Figure 4B-D).

**Table 2:** Correlation between synovial TRAF6 expression and parameters of inflammation or joint destruction in RA patients^▲^

	Intimal TRAF6		Subintimal TRAF6		Total TRAF6	
	
	R	*P*	r	*P*	r	*P*
***Synovial inflammation***						
Synovitis score	0.239	0.119	0.465	0.001**	0.412	0.006**
hyperplasia of intima	0.232	0.130	0.277	0.068	0.269	0.077
Inflammatory cell infiltration	0.164	0.286	0.450	0.002**	0.367	0.014*
Cellular density of the synovial stroma	0.147	0.340	0.118	0.444	0.164	0.288
CD3+ T cells	0.367	0.033*	0.526	0.001**	0.478	0.004**
CD20+ B cell	0.165	0.343	0.359	0.034*	0.313	0.047*
CD38+ plasma cells	0.200	0.250	0.391	0.020*	0.390	0.020**
Subintimal CD68+ cells	0.356	0.074	0.562	0.003*	0.532	0.005**
Intimal CD68+ cells	0.631	0.000***	0.604	0.000***	0.636	0.000***
MCV	0.578	0.005**	0.522	0.013*	0.569	0.006**
CD79a+ B lineage cells	0.154	0.426	0.417	0.024*	0.398	0.032*

***Disease duration***	-0.161	0.298	-0.144	0.352	-0.145	0.347

***Disease activity***						
DAS28CRP	-0.186	0.226	-0.179	0.245	-0.157	0.308
CRP	0.102	0.513	0.103	0.510	0.128	0.413
ESR	-0.133	0.394	-0.058	0.710	-0.057	0.719
RF	-0.094	0.546	-0.003	0.986	0.005	0.975
ACPA	-0.067	0.692	0.110	0.517	0.058	0.733

***Joint destruction***						
Joint space narrowing subscore	-0.182	0.238	-0.103	0.508	-0.106	0.493
Erosion subscore	-0.233	0.128	-0.125	0.419	-0.145	0.347
Total Sharp score	-0.182	0.238	-0.076	0.622	-0.085	0.583

^**▲ **^Spearman's rank order correlation test was used to assess the correlation between synovial TRAF6 expression and the histological, clinical or joint destruction parameters in 44 RA patients.

ACPA, anti-cyclic citrullinated peptide antibody; DAS28, Disease Activity Score 28-joint assessment; CRP, C-reactive protein; ESR, erythrocyte sedimentation rate; MCV, microvascular count;RA, rheumatoid arthritis; RF, rheumatoid factor; *P < 0.05, ** *P *< 0.01, *** *P *< 0.001.

To explore the possible different effect of synovial TRAF6 expression, we subanalyzed intimal and subintimal TRAF6 expression in different grades of synovitis groups and found no significant difference in intimal or subintimal TRAF6 expression between low-grade and high-grade synovitis RA patients. Our data suggested only subintimal TRAF6 expression correlated significantly with synovitis scores (r = 0.465, *P *= 0.001) and the inflammatory cell infiltration (r = 0.450, *P *= 0.002) (Table 2).

### Synovial TRAF6 expression correlated with the density of the common mononuclear inflammatory cells

Sequential slides from those used for TRAF6 staining were then stained for the aforementioned CD markers. In RA synovium, intimal CD68+ cells, cell density of subintimal CD68+ cells, CD3+ cells and CD34+ cells were significantly higher in the high TRAF6 expression group than that in the low TRAF6 expression group. Moreover, significant correlation of TRAF6 expression was detected with intimal CD68+ cell density (r = 0.636, *P *< 0.0001), and subintimal CD68+ cell density (r = 0.532, *P *= 0.005), CD3+ cell density (r = 0.478, *P *= 0.004), CD20+ cell density (r = 0.313, *P *= 0.047), CD38+ cell density (r = 0.390, *P *= 0.020), CD79a+ cell density (r = 0.398, *P *= 0.032) as well as MCV (r = 0.596, *P *= 0.006) (Table 2).

When intimal and subintimal TRAF6 expression were subanalyzed, intimal CD68+ cells and subintimal CD3+ cell density were shown to be correlated significantly with intimal TRAF6 expression (r = 0.631 and 0.367, *P *< 0.0001 and *P *= 0.033, respectively). However, subintimal TRAF6 expression correlated significantly with subintimal CD3+ cell density (r = 0.526, *P *= 0.001), CD20+ cell density (r = 0.359, *P *= 0.034), CD38+ cell density (r = 0.391, *P *= 0.020), CD79a+ cell density (r = 0.417, *P *= 0.024), CD68+ cell density (r = 0.562, *P *= 0.003) and MCV (r = 0.522, *P *= 0.013), as well as intimal CD68+ cell density (r = 0.604, *P *< 0.0001) (Table 2).

In addition, the density of subintimal common mononuclear inflammatory cell types, including CD68+ cells, CD3+ cells, CD38+ cells, CD20+ cells, and CD79a+ cells, were significantly higher in high-grade synovitis RA patients than that in low-grade synovitis RA patients. Moreover, the density of subintimal CD68+ cells, CD3+ cells, CD38+ cells, CD20+ cells and CD79a+ cells correlated positively with the synovitis score (r = 0.306, 0.539, 0.635, 0.752, and 0.771, respectively; *P *= 0.028, *P *= 0.001, *P *< 0.0001, *P *< 0.0001, and *P *< 0.0001, respectively). Therefore, subintimal common mononuclear inflammatory cells may be useful indicators of histologic disease activity in RA.

### Correlation of synovial TRAF6 expression with clinical parameters

Spearman's rank order correlation test was performed to investigate the correlation between synovial TRAF6 expression and ESR, CRP, RF, ACPA, TJC in 28 joints, SJC in 28 joints, HAQ, morning stiffness, gripping power or DAS28-CRP, all of which are serological or clinical parameters that reflect disease activity or severity of RA. No significant correlation was found between synovial TRAF6 expression and DAS28-CRP (*P *= 0.308) in RA. None of the above parameters correlated significantly with synovial TRAF6 expression (Table 2). No significant correlation was found between the presence/absence of synovial TRAF6 expression and age or gender or disease duration. Moreover, the above parameters were not different significantly between low TRAF6 expression group and high TRAF6 expression group.

In addition, about 80% of RA patients in our study were positive for RF and 84% were positive for ACPA, and synovial TRAF6 expression were similar in seropositive and seronegative patients (data not shown).

### Correlation of synovial TRAF6 expression with joint destruction

Unfortunately, no significant correlation was found between synovial TRAF6 expression and total Sharp score (*P *= 0.583), the erosion subscore (*P *= 0.347) or the joint space narrowing subscore (*P *= 0.493) (Table 2). Moreover, total Sharp score, the erosion subscore and the joint space narrowing subscore were not different significantly between low TRAF6 expression group and high TRAF6 expression group. However, disease duration correlated significantly with total Sharp score (r = 0.586, *P *< 0.0001), the erosion subscore (r = 0.533, *P *< 0.0001), and the joint space narrowing subscore (r = 0.562, *P *< 0.0001). Additionally, DAS28-CRP correlated significantly with the erosion subscore (r = 0.325, *P *= 0.032).

### Subanalysis of treated and untreated RA patients

To exclude the influence of therapy on patient recruiting, we further subanalyzed RA patients between treated and untreated group. There was no significant difference in disease duration, age, gender or the age of disease onset between treated and untreated RA patients. There was no significant difference in clinical, serological or radiological parameters related to disease activity or severity, either. Between treated and untreated RA patients, there was no significant difference in total synovial TRAF6 expression, intimal or subintimal TRAF6 expression. But higher TRAF6 expression was found both in treated and untreated RA patients than in OA or Orth.A patients.

Four RA patients treated with methotrexate and etanercept were followed for 12 months and underwent repetitive synovium biopsy. These patients were between 30 and 51 years of age, 1 male and 3 female. Disease activity and pain decreased after 12 months compared to baseline, and synovial TRAF6 expression decreased obviously, paralleled with the decrease of synovitis score, DAS28-CRP, and Sharp score (Table 3). Wilcoxon Signed Ranks Test showed the difference of DAS28-CRP and synovial TRAF6 expression between baseline and 12 months later was at borderline significance (*P *= 0.068 and 0.059, respectively). No significant difference was found in synovitis score and Sharp score between baseline and 12 months later (*P *= 0.257 and 0.141, respectively). As there were only four patients biopsied repetitively after follow up, we presumed there was actually a lack of power to detect this difference in a statistically significant manner.

**Table 3:** Comparative analysis of synovial TRAF6 expression and disease activity in four RA patients during follow-up

	DAS28-CRP (mean)	Total Sharp score (mean)	Synovitis score (mean)	Synovial TRAF6 expression (mean)
Baseline	6.6	25	3	2.8
12 months after treatments	3.2	16	2	1.8

CRP, C-reactive protein; DAS28, Disease Activity Score 28-joint assessment; RA, rheumatoid arthritis; TRAF6, Tumor necrosis factor receptor-associated factor 6

## Discussion

Our study showed that TRAF6 was expressed in most of the intimal cells and obviously expressed in CD68+ cells and some other CD68- cells in the subintimal area, and TRAF6 expression in RA synovium was significantly higher than that in OA or Orth.A synovium. Moreover, synovial TRAF6 expression has significant correlation with histological synovitis score and cell density of subintimal mononuclear inflammatory cells including CD3+ cells, CD38+ cells, CD20+ cells, CD68+ cells and CD79a+ cells in RA. Thus, we postulated that elevated synovial TRAF6 expression may be involved in the pathogenesis of RA synovial inflammation.

RA is a chronic inﬂammatory disease characterized by articular cartilage and bone destruction following growth of the inﬂamed synovial tissue over the articular surface. The pathogenesis of RA depends on a number of different cell types, including macrophages, which are the primary source of the proinflammatory cytokines, as well as dendritic cells, T cells, B cells, plasma cells, endothelial cells, synoviocytes and neutrophils, which produce a panoply of proinﬂammatory cytokines, chemokines, growth factors, matrix metalloproteinases (MMPs) and other proteolytic enzymes that degrade the articular matrix [35,36]. In addition, in the pathogenesis process of RA, osteoclast precursors are triggered to differentiate into mature osteoclasts, which swing the balance of normal bone turnover in favor of net resorption [37]. Our previous study [7] and the present study showed the density of subintimal mononuclear inflammatory cells correlated positively with the synovitis score, which confirmed that different cell types, especially inflammatory cells, involved in the pathophysiology of RA.

IL-1β and TNF-α play important roles in the communication among many cells in the rheumatoid joint [38]. TRAF6 functions as a common and critical signaling adapter molecule acting at the downstream of the IL-1R/TLR family and TNFR superfamily [39]. TLRs have been considered to play essential roles in the pathogenesis of RA, and certain TLRs, such as TLR2, 3, 4 and 7, exhibit a high expression in RA synovium [40,41]. It is also shown that activation by endogenous TLR ligands might contribute to the persistent expression of proinflammatory cytokines by macrophages and the joint damage to cartilage and bone that occurs in RA [42]. Therefore, we suspected that TRAF6 was aberrant in RA synovium, and aimed to reveal the pathophysiological role of TRAF6 in RA. In the present study, we found intense staining of TRAF6 in the endochylema, as well as in the nucleus of intimal synoviocytes and subintimal inflammatory cells of RA synovium, which was significantly higher than that of the OA or Orth.A synovium. Additionally, synovial TRAF6 expression correlated significantly with the synovitis score and the inflammatory cell infiltration. Recruitment and retention of inﬂammatory cells is a fundamental process in synovitis [43]. Here we showed that the cell density of subintimal CD68+ cells, CD3+ cells and CD34+ cells in the high TRAF6 expression group of RA synovium were significantly higher than that in the low TRAF6 expression group, and synovial TRAF6 expression was correlated signiﬁcantly with cell density of subintimal mononuclear inflammatory cells, including CD68+ cells, CD3+ cells, CD38+ cells, CD20+ cells and CD79a+ cells. We then postulated that aberrant synovial TRAF6 expression may be involved in the pathogenesis of RA synovial inflammation. Ahonen C *et al. *reported TRAF6 played a very important role in triggering terminal B cell differentiation to plasma cells, and the loss of TRAF6 recruitment abrogated the generation of long-lived bone marrow plasma cells that secrete either low- or high-affinity nitrophenol-specific antibodies [44]. Here we also found intense staining of TRAF6 in plasma cells located at the subintimal layer of the RA synovium, which implied that TRAF6 may be involved in differentiation and antibody secretion of plasma cells in RA.

To exclude the influence of therapy on patient recruiting, we subanalyzed RA patients between treated and untreated group and no significant difference was found on all demographic and clinical parameters related to disease activity or severity. There was no significant difference on total synovial TRAF6 expression, intimal or subintimal TRAF6 expression between treated and untreated active RA patients, either. But higher TRAF6 expression was found both in treated and untreated RA patients than that in OA or Orth.A patients. Repetitive synovium biopsy was used to investigate the influence of therapy. Synovial TRAF6 expression decreased in parallel with improvement of the clinical disease activity, the histologic disease activity and radiographic joint destruction after 12 months' treatment compared to baseline, suggesting that synovial TRAF6 expression might reflect disease activity in RA.

Synovial macrophages are believed to play a crucial role in joint destruction in RA. Y. Fujikawa *et al. *reported synovial macrophages could differentiate into osteoclast-like cells when co-cultured with osteoblast-like UMR 106 cells, and indicated that synovial macrophages, like monocytes, were capable of altering their phenotype to that of osteoclasts when cultured under specific microenviroment [45]. Expression of CD68 is a valuable marker for identifying the monocyte/macrophage lineage cells, and CD68 is also expressed by authentic osteoclasts. Our present study showed obvious TRAF6 expression in intimal and subintimal CD68+ cells, CD68+ cell desity in the high TRAF6 expression group of RA synovium was significantly higher than that in the low TRAF6 expression group, and significant correlation was found between synovial TRAF6 expression and CD68+ cell density. Considering the well documented role of TRAF6 signaling in RANK-mediated osteoclastogenesis [15], it may, therefore, be hypothesized that TRAF6 is involved in differentiation of osteoclasts in RA.

Our previous study had found significant correlation between subintimal CD79a+ cell density and total Sharp score, the erosion subscore, as well as the joint space narrowing subscore in RA [7], but the underlying mechanism has not been clarified. Bone remodeling and bone loss are controlled by the RANKL-RANK- osteoprotegerin (OPG) axis. RANKL expression could be detected in inﬂammatory cells isolated from the synovial ﬂuid of patients with adult or juvenile RA and patients with OA, while OPG was not detectable [46]. RANKL is the trigger of bone loss and crippling in all animal models of arthritis studied so far [47]. Recent phase II clinical trials suggested that inhibition of RANKL in human RA patients prevented bone loss at the site of inﬂammation without apparent effects on inﬂammation [48]. IL-1β is a key link between synovitis and cartilage breakdown [49]. TRAF6 is an important signaling adapter both in the RANK-RANKL signaling pathway and IL-1β/IL-1 receptor signaling pathway, but the role of TRAF6 in RA bone destruction is poorly understood. Here synovial TRAF6 expression in RA patients showed significant correlation with the density of CD79a+ cells, but no significant correlation with total Sharp score, the erosion subscore or the joint space narrowing subscore. We speculated that there might actually be a lack of power, with only 44 RA patients, to detect this difference in a statistically significant manner. There was likely only sufficient power to detect very strong correlations.

## Conclusions

Our studies indicate that increased synovial TRAF6 expression in RA correlated significantly with histological synovitis severity and cell density of subintimal mononuclear inflammatory cells, as well as intimal CD68+ cells. It may, therefore, be hypothesized that synovial TRAF6 may be involved in the pathogenesis of RA synovial inflammation and osteoclast differentiation. TRAF6 may represent a future target for therapy of RA. A potential limitation of the present study was the lack of demonstrated expression of TRAF6 in osteoclasts or their earlier differentiation stages other than CD68+ cells; therefore, further research on subchondral tissues obtained from arthroplasty is needed. As few patients were recruited in this study, more treatment naïve RA patients and patients biopsied repetitively after treatment are needed in the future. It is worthy of further study - using TRAF6 inhibiters or siRNA silencing - to investigate the exact mechanism of how TRAF6 effect local inflammation and joint destruction in RA.

## Abbreviations

ACPA: anti-cyclic citrullinated peptide antibody; CRP: C-reactive protein; DAS28: Disease Activity Score 28-joint assessment; DMARDs: disease-modifying antirheumatic drugs; ESR: erythrocyte sedimentation rate; FLSs: fibroblast-like synoviocytes; H&E: hematoxylin and eosin; HAQ: Health Assessment Questionnaire; hpf: high-power field; IL-1: interleukin-1; IL-1R: IL-1 receptor; LPS: lipopolysaccharide; mAb: monoclonal antibody; MAPK: mitogen-activated protein kinases; MCV: microvascular count; MMPs: matrix metalloproteinases; NF-κB: nuclear factor κB; OA: osteoarthritis; OPG: osteoprotegerin; Orth.A: orthopedic arthropathies; RA: rheumatoid arthritis; RANK: receptor activator of NF-κB; RANKL: receptor activator of NF-κB ligand; RF: rheumatoid factor; SD: standard deviation; SJC: swollen joint count; TJC: tender joint count; TLR: Toll-like receptor; TNF: tumor necrosis factor; TNFR: TNF receptor; TRAF6: tumor necrosis factor receptor-associated factor 6.

## Competing interests

The authors declare that they have no competing interests.

## Authors' contributions

All authors contributed to the final manuscript. LJZ participated in the design of the study, carried out the experiments and statistical analysis, and drafted and revised the manuscript. LD designed the study, collected data, analyzed data, and drafted and critically revised the manuscript. DHZ helped with collection and acquisition of the data and the synovium, and drafted and critically revised the manuscript. YQM, XOY and XNW assisted with carrying out the experiments and collecting data, analyzed data and critically revised the manuscript. JS assisted in scoring radiographs, performed the statistical analysis and critically revised the manuscript. BYZ helped with collection of the clinical materials, analyzed data and critically revised the manuscript. All authors read and approved the final manuscript for publication.
